# Neurodevelopmental evaluation of newborns who underwent hypothermia with a diagnosis of hypoxic ischemic encephalopathy based on the Bayley-III scale

**DOI:** 10.55730/1300-0144.5748

**Published:** 2023-11-04

**Authors:** Mehmet Fatih DEVECİ, Şenay GÜVEN BAYSAL, Meral ALAGÖZ, İsmail Kürşad GÖKÇE, Derya GÜMÜŞ DOĞAN, Ramazan ÖZDEMİR

**Affiliations:** 1 Neonatal Intensive Care Unit, Şanlıurfa Training and Research Hospital, Şanlıurfa, Turkiye; 2 Developmental Pediatrics Unit, Gazi Yaşargil Training and Research Hospital, Diyarbakır, Turkiye; 3 Division of Neonatology, Department of Pediatrics, İnönü University School of Medicine, Malatya, Turkiye; 4 Division of Developmental Pediatrics, Department of Pediatrics, İnönü University Faculty of Medicine, Malatya, Turkiye

**Keywords:** Hypoxic ischemic encephalopathy, Bayley-III, therapeutic hypothermia, neurodevelopmental evaluation

## Abstract

**Background/aim:**

Hypoxic ischemic encephalopathy (HIE) is one of the common causes of mortality and morbidity in newborns. Despite therapeutic hypothermia, an important treatment with proven efficacy, the morbidity and mortality rates remain high. The aim of this study was to neurodevelopmentally evaluate patients who underwent therapeutic hypothermia.

**Material and method:**

Included herein were patients who underwent hypothermia between 2018 and 2020. Their medical files were reviewed retrospectively, and their demographic and clinical information was recorded. Patients whose contact information was available were called to the developmental pediatrics outpatient clinic for a neurodevelopmental evaluation. The Bayley Scales of Infant and Toddler Development 3rd Edition (Bayley-III) was used as the evaluation tool. Laboratory values and clinical parameters of the patients were further analyzed.

**Results:**

It was found that 42 patients underwent hypothermia in 3 years, of whom 14 (33.3%) had died. Of the 28 patients who were discharged, 20 children could be reached, and a neurodevelopmental evaluation was performed. Developmental delay in the cognitive area was detected in 11 (55%) patients, delay in the language area was found in 9 (45%) patients, and delay in the motor area was found in 11 (55%) patients. The correlation and regression analysis results determined that the time to start cooling was the most effective common factor in all 3 fields of scoring.

**Conclusion:**

The time to start cooling is related to the neurodevelopmental outcomes of patients with HIE. The earlier cooling is started, the better the neurodevelopmental results. Despite therapeutic hypothermia, the neurodevelopmental development of infants may be adversely affected. These patients should be followed-up neurodevelopmentally for a long time.

## 1. Introduction

Hypoxic ischemic encephalopathy (HIE), one of the common causes of death and disabilities in newborns, is an acute progressive encephalopathy that occurs in infants exposed to intrapartum or postpartum hypoxia [1]. Today, despite the developments in the care of newborn babies, the incidence of HIE is 1.5–2 per 1000 live births [2]. Every year, 7500 babies are exposed to moderate or severe HIE around the world, and of these infants, 400,000 have neurodevelopmental issues [3]. Motor and behavioral damage such as cerebral palsy, visual and auditory problems, epilepsy, global developmental delay, and autism can be seen [4–6]. Neuroprotective treatments should be initiated as soon as possible to reduce the mortality and sequelae rates of infants exposed to hypoxic ischemia. Hypothermia is the only option that has been proven to improve neurodevelopmental outcomes among neuroprotective treatments [7]. In randomized studies, hypothermia treatment has been reported to reduce the mortality rate and the risk of neurodevelopmental damage in patients with HIE. Even with hypothermia treatment, patients who survive HIE can show severe neurodevelopmental problems such as cerebral palsy and mental disability [8–10]. In addition, the risk of minor neurodevelopmental issues (difficulty in reading and arithmetic skills, attention deficit-hyperactivity, behavioral and social adjustment difficulties) increases in these children [11,12].

HIE is a significant cause of death in newborns. Acute neurological damage in the early period and permanent neurodevelopmental problems in the long term can be seen in the survivors. Patients with HIE should be followed-up in the long term in terms of neurodevelopment. Early neurodevelopment evaluation is critical because it allows for earlier intervention by detecting developmental problems. The Bayley Scales of Infant and Toddler Development 3rd Edition (Bayley-III), the gold standard around the world, is a good tool for detecting problems in these patients [13]. In this study, a neurodevelopmental evaluation of the patients who underwent hypothermia in our neonatal intensive care unit, between 2018 and 2020, was performed by applying the Bayley-III. The Bayley-III is a specialized, up-to-date, and globally accepted development test that requires expertise. In the literature, there are a limited number of neurodevelopmental evaluation studies conducted with the Bayley-III in a patient group with HIE. Thus, it was aimed herein to emphasize the neurodevelopmental evaluation with the Bayley-III and long-term follow-up of HIE patients.

## 2. Material and methods

### 2.1. Patient selection

This study began after receiving approval ethics. The medical files of patients admitted to our neonatal intensive care unit between January 2018 and December 2020 were scanned. Demographic characteristics (birth weight, place of birth, delivery method, gestational week, and sex), laboratory tests (blood gas taken from the cord or within the first postnatal hour, kidney and liver function tests taken at 24 h), seizure history, amplitude-integrated electroencephalogram (aEEG) follow-ups, and discharge information were obtained retrospectively from the medical files of HIE patients who received hypothermia treatment. After accessing the contact information for the parents of the surviving patients, they were called and asked to bring the patients to the developmental pediatrics outpatient clinic for a neurodevelopmental evaluation. The development of the patients whose parents agreed to participate in the study were evaluated with the Bayley-III. An experienced developmental pediatrician performed the Bayley-III, and the evaluations of each patient lasted approximately 90–150 min.

### 2.2. Hypothermia protocol

Hypothermia treatment was applied to the patients who met clinical and/or laboratory indications. The indications for hypothermia treatment for patients in our unit were [1,14]: 1) having a gestational age ≥36 weeks and within the first 6 h postnatally, 2) a blood gas pH <7.00 or a base deficit less than −16 mmol/L when taken within the first hour from the cord or postnatally, 3) an appearance, pulse, grimace, activity, and respiration (APGAR) score <5 or an ongoing need for resuscitation in the 10th min, 4) moderate and severe HIE according to Sarnat staging on clinical evaluation, 5) moderate or severe trace disorder if aEEG is used, 6) low APGAR or encephalopathy findings in those with a pH of 7.00–7.15 and a base excess (BE) of 10–16.

Hypothermia treatment was applied to all of the patients using a Tecotherm Neo Infant Cooling System (Inspiration Healthcare Ltd., Croydon, England, UK). During the treatment, the patients were monitored. The core temperatures of the patients were monitored with a rectal heat probe and kept at 33.5–34.5 °C for 72 h.

### 2.3. The Bayley-III

The Bayley-III was updated by Nancy Bayley in the USA in 2006. The Bayley-III is a gold standard assessment scale that measures 5 different developmental areas (cognitive, expressive language, receptive language, fine motor, and gross motor) in 1–42-month-old children. The scale was standardized with a sample of 1700 children in the USA, with high reliability (0.80–0.87) and validity (0.86–0.93). It provides a better understanding of early development in the high-risk group and delivers more sensitive results for clinical trials. Bayley-III test results were expressed as developmental area indices, an average of 100 points, and a standard deviation (SD) of 15 points. For developmental areas, composite scores of 70 to 84 (SD of −1 to −2) are considered abnormal, and composite scores lower than 69 (SD less than −2) indicate severe developmental delay [13].

### 2.4. Statistical analysis

The conformity of the variables to normal distribution was examined using the Shapiro–Wilk test. Continuous variables were expressed as the mean ± SD in the case of compliance with normal distribution and as median (minimum–maximum) values in the absence of normal distribution. Categorical variables were expressed as n (%) values. According to the normality test result, the Independent Sample’s t test and Mann–Whitney U test were used to compare the 2 groups. The chi-squared test, Fisher s precise chi-squared test, and Fisher–Freeman–Halton test were used to analyze the categorical variables. The relationship between the continuous variables was examined using the Pearson correlation coefficient and Spearman correlation coefficient. The multiple linear regression analysis backward method was used to determine the factors affecting the language, cognitive, and motor values. IBM SPSS Statistics for Windows 21.0 (IBM Corp., Armonk, NY, USA) was used and p < 0.05 was considered statistically significant.

## 3. Results

Hypothermia was applied to 42 patients between January 2018 and December 2020. Of these patients, 19 were female. The mean birth weight was 3227.62 ± 511.97 g and the median gestational week was 39 (35–42). Of these patients, 14 died, and the mortality rate was 33.3%. When the patients were evaluated according to the Sarnat staging, 26 were classified as having moderate HIE and 16 had severe HIE. First, the patients were divided into moderate and severe groups according to their staging. Then, the demographic, laboratory, and clinical data between the groups were analyzed. There was no difference between the groups regarding the demographic data (birth week, birth weight, sex, and asphyxia etiology). It was found that the number of patients with severe HIE who had seizures and an abnormal aEEG trace was higher, the median duration of resuscitation was longer, the mortality rate was higher, and the mean APGAR score was lower (Table 1). In the laboratory data of the patients, low PH, high BE, renal dysfunction, and high transaminase levels were statistically significant in the patients with severe HIE (Table 2).

Neurodevelopmental evaluation was performed in 20 of the 28 patients discharged from the neonatal intensive care unit. Of these patients, 16 were in the moderate HIE group and 4 were in the severe HIE group. The patients were evaluated neurodevelopmentally at an average of 21.65 ± 8.72 months. The results showed that the mean cognitive composite score was 77.55 ± 14.31, the mean language composite score was 84.4 ± 17.57, and the mean motor composite score was 80.05 ± 16.89. In all 3 field evaluations, the composite scores of those with severe HIE were lower (Table 3).

It was determined that 11 patients with cognitive composite scores <85 had lower pH and delayed cooling initiation time, and this difference was statistically significant (p = 0.020 and p = 0.007, respectively); 9 patients with language composite scores <85 had lower pH, higher BE, and delayed cooling initiation time, and these differences were statistically significant (p = 0.031, p = 0.012, and p = 0.007, respectively); and 11 patients with motor composite scores <85 had statistically significant low pH, high BE, and delayed cooling initiation time (p = 0.046, p = 0.046, and p = 0.046, respectively).

As a result of the correlation analysis, a significant positive relationship was found between the language composite score and the pH value (p = 0.027). With an increase in the pH value, the language composite score also increased. There was a significant negative correlation between the language composite score and the BE, uric acid, creatinine, and cooling initiation values (p = 0.013, p = 0.021, p = 0.042, and p = 0.003, respectively). The highest correlation was between the cooling initiation time and the language composite score. An increase in the BE, uric acid, creatinine, and cooling initiation time caused a decrease in the language composite score. There was a significant positive relationship between the cognitive composite score and the pH value (p = 0.008). With an increase in the pH value, the cognitive composite score also increased. There was a significant negative correlation between the cognitive composite score and the cooling initiation time and BE value (p = 0.002, p = 0.013). As the cooling initiation time increased, the cognitive composite score decreased. There was a significant positive relationship between the motor composite score and the pH value (p = 0.014). As the pH value increased, the motor composite score also increased. There was a significant negative correlation between the motor composite score and the BE, creatinine, and cooling initiation values (p = 0.023, p = 0.011, and p = 0.007, respectively). An increase in the BE, creatinine, and cooling initiation values caused a decrease in the motor composite score (Table 4).

In the regression analysis, the uric acid value and cooling initiation time were determined as influential factors on the language composite score (p = 0.017 and p = 0.003, respectively). A 1-unit increase in the uric acid value caused a 3.234-unit decrease in the language composite score, and a 1-unit increase in the cooling initiation time caused a 9.09-unit decrease in the language composite score. According to the analysis results, the model established was found to be significant (R^2^ = 0.502, p = 0.001). The BE and cooling initiation time were determined as influential factors on the cognitive composite score (p = 0.038 and p = 0.012, respectively). A 1-unit increase in the BE caused a 0.901-unit decrease in the cognitive composite score, and a 1-unit increase in the cooling initiation time caused a 6.647-unit reduction. According to the analysis results, the model established was found to be significant (R^2^ = 0.484, p = 0.002). The creatinine and cooling initiation values were determined as influential factors on the motor composite score (p = 0.030 and p = 0.021, respectively). A 1-unit increase in the creatinine value caused a 33.055-unit decrease in the motor composite score, and a 1-unit increase in the cooling initiation time caused a 7.475-unit decrease. According to the analysis results, the model was significant (R^2^ = 0.460 p = 0.003) (Table 5).

## 4. Discussion

In this study, it was determined that patients diagnosed with HIE could be affected neurodevelopmentally, despite hypothermia treatment, and the earlier hypothermia was initiated, the higher the Bayley-III scores. It is recommended that hypothermia is initiated within the first 6 h, before entering the irreversible latent period. Studies have shown that the earlier the initiation, the better the efficacy. Thoresen et al. [15] found that hypothermia treatment initiated in the first 3 h was more reliable. Although hypothermia was initiated in all of the patients in the present cohort within the first 6 h, the cooling initiation time was determined to be the common factor affecting all 3 development area scores of the patients. The current study determined that the cooling initiation time was the most effective factor in the scoring of all 3 areas in the correlation and regression analyses.

Despite the only proven treatment regimen, therapeutic hypothermia, HIE remains a common cause of neonatal mortality and morbidity. Using clinical staging and laboratory evaluations in the first postnatal hours, mortality and sequelae in patients with HIE can be predicted. The likelihood of multiple organ failure increases with the severity of HIE. In some studies, liver and kidney dysfunction were more common in severe HIE and were associated with mortality [16,17]. Again, the level of acidosis in the first blood gas after birth, time of resuscitation, clinical seizure, and an abnormal aEEG trace are also associated with the HIE severity and mortality in these patients [18,19]. The laboratory and clinical data of the patients herein were consistent with the literature.

The first year of human life is critical in terms of cognitive, motor, and visual development. The integrity of motor, sensory, and cognitive development is fundamental for normal neurodevelopment. A lack of sufficient stimuli and deterioration of this integrity negatively affect neurodevelopment and are considered disabilities to varying degrees [20,21]. Although various treatment regimens are currently being sought for newborns diagnosed with HIE, the most vulnerable group for neurodevelopmental difficulties, therapeutic hypothermia remains the only proven treatment regimen [7]. In a randomized study investigating the long-term outcomes of therapeutic hypothermia, 190 children were evaluated at 6–7 years of age, and it was found that their IQ scores were higher, and the mortality rates was lower in those who received hypothermia than in those who did not [22].

The Bayley-III has been used to evaluate the neurodevelopmental status in many groups, such as those who were premature, with critical congenital heart disease, and with Down syndrome [23–25]. Mansson et al. [23] found that the sensitivity to detect children with an IQ of <70 was 18%, and the false-positive rate was 7% when a Bayley-III score of 70 was used as the threshold. When a score of 85 was used, it corresponded to sensitivity and false-positive rates of 44% and 7%, respectively. They supported using a cut-off value of 85 to suspect a subnormal IQ. Finder et al. [26] used the Bayley-III in their study with 690 children and used 85 as the cut-off value. They found that children with mild HIE at the age of 2 had lower cognitive compound scores than the healthy control group. A cut-off value of 85 was used herein, and those with a score <85 were accepted as neurodevelopmentally delayed. Herein, patients with a score <85 had a lower pH and a later cooling initiation time.

In a systemic review examining the effects of early interventions on motor development, it was emphasized once again that the Bayley evaluation scale is the most frequently used method to measure neuromotor and developmental outcomes. The structure of the Bayley-III provides more helpful information in understanding early development, improving our ability to identify certain developmental deficiencies. In terms of research, it provides a better understanding of early development in the high-risk group and provides more sensitive results for clinical studies [27,28]. Bode et al. [29] showed that 2-year-old Bayley-III cognitive and language scores in preterm children had a robust guiding value for 4-year-old IQ. In a study conducted in Türkiye, it was emphasized that early neurodevelopmental evaluation will be a guide for long-term follow-ups and is essential in terms of monitoring and supporting the development of infants [30]. The patients herein were evaluated neurodevelopmentally at an average of 21.6 months and long-term neurodevelopmental follow-ups were planned for all them.

The current study was conducted on patients with moderate and severe HIE. Today, hypothermia is required for these patients. The study was limited by the lack of a control group without hypothermia, the fact that it was a single-center study, and the small sample size. Furthermore, using a valuable scale like the Bayley-III strengthened the research findings.

In conclusion, multiple organ failure, acidosis depth and resuscitation time, and clinical seizures are all associated with the degree of HIE, according to the findings herein. These will have a predictive value in the neurodevelopmental follow-up of patients. Despite hypothermia, neurodevelopmental effects may occur in patients. These patients should be followed-up neurodevelopmentally for a long time. The earlier the hypothermia treatment is initiated, even within the recommended first 6 h, the better the neurodevelopmental results will be.

## Figures and Tables

**Table 1:** Demographic and clinical characteristics of the patients who underwent hypothermia.

	All patients (n = 42)	Moderate HIE (n = 26)	Severe HIE (n = 16)	p-value
Sex (female) n (%)	19 (45.2)	13 (50)	6 (37.5)	0.429^a^
Gestational age, weeks	39 (35–42)	39 (35–42)	39 (35–41)	0.240^e^
Birth weight, g	3227.62 ± 511.97	3206.15 ± 434.43	3262.5 ± 632.29	0.734^d^
Referred from other centers, n (%)	39 (92.9)	24 (92.3)	15 (93.8)	>0.99^b^
Mode of delivery (caesarean), n (%)	19 (45.2)	13 (50)	6 (37.5)	0.429^a^
Maternal age, years	30.17 ± 6.57	30.04 ± 5.23	30.38 ± 8.49	0.888^d^
10th-min APGAR score	5.17 ± 2.17	6 ± 1.81	3.81 ± 2.07	**0.001** d
Resuscitation time, min	1 (0–20)	1 (0–20)	4 (15–42)	**0.028** e
Active cooling initiation time, h	3 (1–10)	3 (1–10)	3 (1–5.5)	0.701^e^
Abnormal aEEG trace, n (%)	18 (42.9)	6 (23.1)	12 (75)	**0.001** a
Seizures in the first 72 h, n (%)	23 (54.8)	20 (76.9)	3 (18.8)	**<0.001** a
Distribution of etiology				
Meconium aspiration syndrome, n (%)	11 (26.2)	7 (26.9)	4 (25)	0.134^c^
Difficult delivery/prolonged labor, n (%)	20 (47.6)	15 (57.7)	5 (31.2)	
Cord entanglement, n (%)*	3 (7.1)	2 (7.7)	1 (6.3)	
Cord prolapse, n (%)*	2 (4.8)	1 (3.8)	1 (6.3)	
Fetal distress, n (%)*	5 (11.9)	0	5 (31.2)	
Placental abruption, n (%)*	1 (2.4)	1 (3.8)	0	
Mortality rate, n (%)	14 (33.3)	4 (15.4)	10 (62.5)	**0.002** a

Mean ± SD, median (minimum–maximum) and n (%) values are given in the definitions of the variables.

* Combined and analyzed due to an insufficient number of samples.

a: Chi-squared test,

b: Fisher’s exact chi-squared test,

c: Fisher Freeman–Halton test,

d: Independent Samples t-test,

e: Mann–Whitney U test.

**Table 2:** First blood gas and 24-h laboratory parameters of all of the patients who underwent hypothermia. Mean ± SD and median (minimum–maximum) values are given in the definitions of the variables. d: Independent Samples t test, e: Mann–Whitney U test.

	All patients (n = 42)	Moderate HIE (n = 26)	Severe HIE (n = 16)	p-value
Ph	6.90 (6.5–7.1)	6.94 (6.8–7.1)	6.88 (6.5–6.99)	**0.002** ^e^
Partial pressure of carbon dioxide mmHg	62.29 ± 26.31	55.72 ± 13.84	72.55 ± 36.81	0.097^d^
Bicarbonate mmol/L	9.38 ± 2.67	10.55 ± 2.24	7.49 ± 2.25	**<0.001** ^d^
Base excess mmol/L	−20.60 ± 5.36	−18.93 ± 4.26	−23.23 ± 5.95	**0.010** ^d^
Lactate mmol/L	14.33 ± 5.15	13.23 ± 4.62	16.04 ± 5.61	0.088^d^
White blood cells 10^3/UL	22.91 ± 8.51	19.79 ± 7.23	27.98 ± 8.17	**0.002** ^d^
Hemoglobin g/dL	17.13 ± 3.02	17.46 ± 2.89	16.58 ± 3.24	0.363^d^
Platelet 10^3/UL	216.57 ± 81.27	226.46 ± 78.04	200.5 ± 86.37	0.321^d^
Blood urea nitrogen mg/dL	12.31 ± 4.22	11.47 ± 3.96	13.71 ± 4.41	0.104^d^
Creatinine mg/dL	0.90 (0.41–2.0)	0.84 (0.5–2)	0.97 (0.41–1.7)	**0.036** ^e^
Uric acid mg/Dl	8.37 ± 2.57	7.62 ± 2.24	9.72 ± 2.65	**0.012** ^d^
Lactate dehydrogenate U/L	1816.50 (880–11147)	1778 (897–11147)	2933 (880–6000)	0.865^e^
Aspartate aminotransferase U/L	186 (72–4500)	148 (76–4078)	337 (72–4500)	0.065^e^
Alanine aminotransferase U/L	45.5 (15–4200)	38 (15–946)	119 (23–4200)	0.078^e^

**Table 3:** Scoring of the patients evaluated with the Bayley-III.

	All patients (n = 20)	Moderate HIE (n = 16)	Severe HIE (n = 4)	p-value
Language composite score	84.4 ± 17.57	88.94 ± 11.90	66.25 ± 26.42	**0.016** d
Cognitive composite score	77.55 ± 14.31	82.25 ± 11.70	58.75 ± 4.79	**<0.001** d
Motor composite score	80.05 ± 16.89	86.13 ± 11.48	55.75 ± 12.82	**<0.001** d

Mean ± SD is given in the definition of the variables.

d: Independent Samples t-test.

**Table 4:** Correlation analysis results of the language, cognitive, and motor scores.

		Language scores (n = 20)	Cognitive scores (n = 20)	Motor scores (n = 20)

Ph	r	**0.493** *	**0.574** **	**0.539** *

	p	**0.027**	**0.008**	**0.014**

BE	r	**0.450** *	**0.543** *	**0.505** *

	p	**0.046**	**0.013**	**0.023**

Lactate	r	−0.159	−0.316	−0.140

	p	0.503	0.174	0.557

Aspartate aminotransferase	r_s_	−0.169	−0.427	−0.271

	p	0.478	0.060	0.249

Alanine aminotransferase	r_s_	−0.132	−0.357	−0.284
	p	0.580	0.122	0.226

Uric acid	r	−0.512*	−0.230	−0.321
	p	**0.021**	0.330	0.168

Creatinine	r	−0.470*	−0.329	−0.569*
	p	**0.042**	0.169	**0.011**

Active cooling initiation time	r	−0.635**	−0.656**	−0.587**
	p	**0.003**	**0.002**	**0.007**

r: Pearson correlation coefficient, rs: Spearman correlation coefficient,

* 0.05

** 0.01 significance level.

**Table 5:** Factors affecting the language, cognitive, and motor scores and the regression analysis results.

Language score	β	SD	T	p-value	95% CI	
					Low	High
Uric acid	−3.234	1.217	−2.657	**0.017**	−5.815	−0.653
Active cooling initiation time	−9.090	2.650	−3.430	**0.003**	−14.708	−3.472
Fix	137.943	12.686	10.873	**<0.001**	111.050	164.837
Model R^2^ = 0.502						
Model significance = 0.001						
Cognitive score	β	SD	T	p-value	95% CI	
					Low	High
BE	0.901	0.398	2.265	**0.038**	0.058	1.744
Active cooling initiation time	−6.647	2.334	−2.847	**0.012**	−11.596	−1.698
Fix	116.554	9.541	12.217	**<0.001**	96.328	136.779
Model R^2^ = 0.484						
Model significance = 0.002						
Motor scores	β	SD	T	p-value	95% CI	
					Low	High
Creatinine	−33.055	13.878	−2.382	**0.030**	−62.475	−3.634
Active cooling initiation time	−7.475	2.931	−2.550	**0.021**	−13.689	−1.262
Fix	133.496	13.478	9.905	**<0.001**	104.924	162.068
Model R^2^ = 0.460						
Model significance = 0.003						

The backward method was used in the regression analysis.

## Data Availability

The data sets used and/or analyzed during the current study are available from the corresponding author on reasonable request.
